# An efficient hybrid method for 3D to 2D medical image registration

**DOI:** 10.1007/s11548-022-02624-0

**Published:** 2022-04-18

**Authors:** Shabnam Saadat, Diana Perriman, Jennie M. Scarvell, Paul N. Smith, Catherine R. Galvin, Joseph Lynch, Mark R. Pickering

**Affiliations:** 1 The School of Engineering and Information Technology, The University of New South Wales, Canberra, Australia; 2 The Trauma and Orthopaedic Research Unit, The Canberra Hospital, Canberra, Australia; 3 The Faculty of Health, University of Canberra, Canberra, Australia; 4 The School of Medicine, The Australian National University, Canberra, Australia; 5 College of Engineering and Computer Science, The Australian National University, Canberra, Australia

**Keywords:** Edge position difference, Image registration, medical image analysis, Similarity measure, Sum-of-conditional variance

## Abstract

**Purpose:**

The purpose of this paper is to present a method for registration of 3D computed tomography to 2D single-plane fluoroscopy knee images to provide 3D motion information for knee joints. This 3D kinematic information has unique utility for examining joint kinematics in conditions such as ligament injury, osteoarthritis and after joint replacement.

**Methods:**

We proposed a non-invasive rigid body image registration method which is based on two different multimodal similarity measures. This hybrid registration method helps to achieve a trade-off among different challenges including, time complexity and accuracy.

**Results:**

We performed a number of experiments to evaluate the performance of the proposed method. The experimental results show that the proposed method is as accurate as one of the most recent registration methods while it is several times faster than that method.

**Conclusion:**

The proposed method is a non-invasive, fast and accurate registration method, which can provide 3D information for knee joint kinematic measurements. This information can be very helpful in improving the accuracy of diagnosis and providing targeted treatment.

## Introduction

Image registration is a fundamental task used to match two or more pictures taken, for example, at different times, from different sensors, or from different viewpoints [4]. In medical image analysis, there is a need for dynamic 3D images of anatomical structures of the human body. This can enable specialists to track events, carry out and evaluate surgical, and radio therapeutical procedures [10]. Roentgen stereo photogrammetry analysis (RSA) [16] is one of the current techniques that is used to measure joint kinematics. This method is accurate, but invasive, because it requires the implanting of tantalum beads into the bone before the image capturing process. Although non-invasive video/optical tracking systems can be used for motion measurements these systems suffer from low accuracy, and the markers used on the skin may move independently of the underlying bone  [18]. Consequently, because of the importance of providing dynamic 3D images, techniques involving 3D to 2D image registration are now being applied in this area. In 3D to 2D image registration, 3D motion information is provided by registering dynamic 2D images with a high-resolution 3D image or a 3D model of the human anatomical structures. In 3D to 2D image registration, multimodal similarity measures [3, 11, 15, 21, 22] should be applied for images captured using different modalities because the relationship between the pixels in the images is nonlinear [11]. Mutual information (MI) is a popular similarity measure which has proven to be a very robust and reliable similarity measure for intensity-based registration of multimodal images, but it is sensitive to the dimensions of overlapped image regions. Studholme in [17] used a normalized mutual information (NMI) measure which addressed this issue. The other difficulty with regard to MI is the registration of small images as the use of prior information estimated from the entire image may lead to false maxima in the MI goal function. To solve this issue, Andronache in [2] applied MI for global registration, and the cross correlation to register small image patches. A combination of NMI and gradient information was also employed in [13]. In [14], a non-rigid image registration approach was proposed. This approach used regional mutual information (RMI) which is an entropy based similarity measure that considers local neighbourhood information. A model-based registration method was proposed in [20]. This method applies a similarity measure based on a weighted edge-matching score (WEMS) which gives a high priority to longer edges in the images to be registered. However, the computation time is still required to be reduced in this method. In  [5] a registration method was proposed in which a weighted histogram of image gradient directions (WHGD) was adopted as the image feature. This method is faster and more robust against large initial displacements compared to existing techniques. However, it may not provide accurate results if there is an irrelevant object in the reference image, and in this case the method requires a pre-registration segmentation. A gradient-based method was applied to register CT to fluoroscopic X-ray images in [7]. In this proposed method, outliers and foreign objects are removed from the fluoroscopic X-ray images using the volume gradients applied. In another recent approach [11], Pickering proposed a new method that applied the multimodal similarity measure SCV in conjunction with Gauss–Newton optimization. This method was shown to be more accurate and robust as well as more computationally efficient than the technique which used MI proposed by Thevenaz and Unser [19]. Another recent multimodal similarity measure is the EPD [15] which has very low computational complexity. A number of methods [21, 22] combine aspects of both structural and neighbourhood information which offers more robustness and a high level of registration accuracy. The authors in [21] proposed a novel integrated method named CNVS for multimodal brain image registration. In [22], a multimodal registration method was proposed which used regional mutual information (RMI). This method used a combination of features and intensity information to offer a more robust method. Dimensional mismatch, the nonlinear pixel relationship of the multimodal images, image quality in terms of resolution and noise due to the low doses of radiation, high computation time, low accuracy, and not being robust against large initial displacements can be considered as the main challenges of the 3D CT to single-plane fluoroscopy image registration approaches. In this paper, we propose a fast and robust hybrid rigid body registration method which is based on two different multimodal similarity measures: edge position difference (EPD) [15] and sum-of-conditional variance (SCV) [12]. In the proposed method, at first, the EPD is used to perform a coarse registration, which reduces the range of the search space covered in the next step that uses a method based on SCV to register the images accurately. The remainder of the paper is arranged as follows: 3D CT to 2D single-plane Fluoroscopy Image Registration explains, in detail, different steps of our proposed hybrid registration method. The experiments carried out to evaluate the performance of the proposed method and the discussion related to these results are provided in Sec7, and finally the paper is concluded in Sec12.

## 3D CT to 2D single-plane fluoroscopy image registration

In 3D CT to 2D single-plane fluoroscopy image registration, real-time video fluoroscopy images are captured by an image intensifier, and stored as frames in a digital video file. A 2D projection of the 3D CT data for each bone is then registered to the image of the same bone in the fluoroscopy frame. In order to register a 3D CT image (a sensed image) to a 2D fluoroscopy image (a reference image), a number of pre-processing methods should be performed on the input images. As single-plane fluoroscopy images suffer from pincushion distortion partly caused by the curved nature of the image intensifier, at first this distortion in the images should be corrected using the method applied in  [11]. Regarding the 3D CT image, this image is segmented by first performing a simple method based on thresholds and it is followed by a more accurate manual segmentation. For example, in the case of knee joints, the soft tissue is removed from the CT image of the patient, and then it is segmented into two separate images for the femur and tibia. In order to find information about the kinematics of a knee joint, each segmented CT image should be registered independently with the same bone in the fluoroscopy frame. After the pre-processing stage, the corrected fluoroscopy and CT image are the input images in the registration process. The proposed registration method has a number of steps that are repeated iteratively until the best transformation for alignment is reached. The first step in the registration section is dimensional correspondence. In the proposed method, we use digitally reconstructed radiographs (DRRs) [6, 11] which is by far the most common method of producing a simulated X-ray projection image from a CT image using ray-casting. By using a DRR, the 3D CT image is projected to a 2D image which can be registered with each 2D fluoroscopy frame. After that, the similarity measure *S* between these 2D images is computed. When the minimum similarity measure *S* is found, the registration finishes, otherwise, the required transformation change is estimated, and the CT image is transformed according to the changes. This process is repeated until the best alignment is found. In the registration stage, a fast and robust method is proposed. Our proposed method is based on a hybrid registration approach, which is divided into coarse and fine registration steps. In the coarse registration step, the geometric transformation parameters are estimated using the EPD similarity measure  [15], and are updated for the fine registration step. In the final registration step, an accurate registration method based on the SCV similarity measure  [11] is employed which is accompanied by a Gauss–Newton optimization method to finally refine in-plane and out-of-plane parameters representing the transformation needed for the matching between the two images to be registered. These registration steps are explained in more detail in Sec3 and Sec6. As out-of-plane transformation parameters are changed by 3D motions perpendicular to the fluoroscopy imaging plane, finding the value of these parameters is much more complicated than finding the in-plane values. Therefore, the in-plane parameters are estimated first which reduces the processing time required in the next steps.

**Fig. 1:**
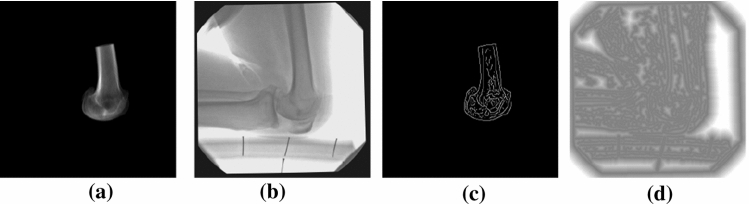
**a**, **b** show an example of 2D projection of a CT image and a fluoroscopy image to be registered. **c** Represents the binary edge image of the 2D projection of the CT image. The distance to the edge position of the fluoroscopy image is shown in **d**

### In-plane registration

In this section, two different methods for finding in-plane parameters are described. In the first in-plane registration method, which is explained in Sec3, an estimation of the in-plane transformation parameters is computed by a fast method based on the EPD similarity measure. In the second method, the in-plane parameters, computed by applying the in-plane fast EPD registration on a wide search range of in-plane parameters, are then used as the input to a more robust but slower in-plane registration method based on the SCV similarity measure. However, this further in-plane registration step, based on the SCV, is applied to a narrower search range. Regarding the initial 3D position of bones to be registered, the first frame is registered semi-automatically. At first, it is registered manually, and then it is registered by the proposed registration method. For the other frames, the registered output which shows the correct position of the bone in the 3D CT image for one frame can be used as the initial position for the 3D CT image for the next frame.

#### In-plane registration based on the EPD similarity measure

In the first step of this in-plane registration method, based on the EPD, the 3D CT volume is segmented, and then projected to a 2D DRR image. Meanwhile, a distortion correction method is applied to the fluoroscopy image. After that, the binary edge images of the fluoroscopy and the 2D DRR images, which are denoted by $E_R$ and $E_I$, respectively, are computed using the Canny edge detection method. This results in a reduction of the amount of data that will be processed in the next steps. Then, a chamfer distance image, denoted by $D_R$, is computed for the fluoroscopy binary edge image. Figure 1 shows an example of using the EPD in the proposed 3D CT to 2D fluoroscopy image registration method. A full search registration algorithm is then performed on a wide range of in-plane parameters ($T_x$, $T_y$ and $R_z$). However, as this method is based on the EPD, which is very fast, the computation time required for this step is quite low. In each iteration of the search on the in-plane parameters, the CT image is transformed according to the new values of the in-plane parameters. After that, the edge position is computed by using the input parameters of the transformed 2D edge image of the DRR ($E_I$) and the chamfer distance computed for the binary edge image of the fluoroscopy ($D_R$). If the EPD similarity measure computed in this iteration is less than the minimum similarity measure $S_{{ min}}$ computed in previous iterations, then the optimal in-plane parameters are updated. These steps are repeated for the entire search range.

#### In-plane registration based on the SCV similarity measure

This in-plane registration method is based on the registration method described in [11] using the SCV similarity measure. SCV is a multimodal similarity measure, which was proposed by Pickering in [12]. This similarity measure is based on the joint probability distribution of the images to be registered. In the first step, the 3D CT volume is segmented and projected to a 2D DRR image. Then, a Laplacian-of-Gaussian (LoG) filter is used on the 2D DRR image. Meanwhile, a distortion correction method is applied on the fluoroscopy image. A LoG filter is then applied to the fluoroscopy image to reduce the amount of noise and enhance the edges. Next, a full search is performed on a range of in-plane parameters. In each iteration of the search, the segmented CT image is transformed according to the new value of the in-plane parameters. After that, the SCV similarity measure is computed using the fluoroscopy and 2D DRR image. If this similarity is less than the minimum similarity computed in previous iterations, the value of the minimum similarity measure $S_{min }$ and optimal in-plane parameters are updated. The whole process is repeated until the maximum similarity between the images to be registered is found.

### Final registration based on the SCV similarity measure

In the final registration step, the hybrid method uses the updated transformation parameters from the previous in-plane registration, explained in Sec3, as the input data. The final registration step is intended to estimate the out-of-plane parameters ($R_x$, $R_y$), and refine and update the in-plane parameters and the out-of-plane translation ($T_z$). Indeed, the final values of all transformation parameters are updated in this stage. The final registration method is based on the registration method in [11] using the SCV similarity measure. The final registration starts with a search which is performed in order to find the out-of-plane rotations ($R_x$ and $R_y$). Inside this search, a Gauss–Newton optimization method is employed to find the best parameters which, when applied to the CT volume, will minimize the similarity measure. Using this optimization, the in-plane parameters and the out-of-plane translation ($T_z$) are refined and updated to provide a more accurate alignment between the input images. To describe the final registration method in more detail, first, the distortion in the fluoroscopy image is corrected, and after that, a LoG filter is applied to the fluoroscopy image. At the same time, the 3D segmented CT image is transformed by the new values of $R_x$ and $R_y$. The transformed image is then projected to a 2D DRR image, and a LoG filter is applied to this image. Then, the SCV similarity measure between the two images (the 2D fluoroscopy and the 2D DRR image), the hessian matrix and the gradient vectors required for the following Gauss–Newton optimization method are computed [11]. Next, in the optimization step, the new changes for $T_x$, $T_y$, $R_z$ and $T_z$ are estimated. Then, the segmented CT is transformed using these new parameters, projected and filtered for a new SCV computation. If the computed SCV is less than the minimum similarity measure computed in the previous steps, the parameters are updated. Then, $R_x$ and $R_y$ are changed and the whole process is repeated for these new values. The optimization method estimates the values of the similarity measure iteratively for each combination of the value of $R_x$ and $R_y$ in a small neighbourhood around the current value of the remaining transformation parameters. This results in an estimation of the changes required for $T_x$, $T_y$, $R_z$ and $T_z$. Finally, the six geometric transformation parameters, which describe the estimated 3D position of the 3D CT volume, form the result of the registration process.

**Fig. 2:**
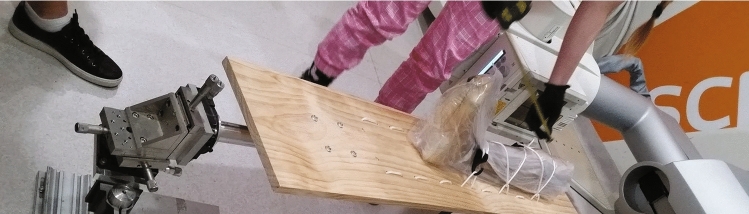
Capturing a fluoroscopy frame from one of the knee cadavers

## Experiments and discussions

To evaluate the performance of the proposed method, a number of experiments were performed to analyse the method’s accuracy and computation time. These experiments are discussed in Sec8 and Sec11. The experiments were run on an Intel Core i7 computer that ran at 3.6 GHz and had 16 GB of RAM. In the experiments, we compared the accuracy and computation time of the proposed algorithm with the registration approach in [11] which is based on the SCV similarity measure optimized by the standard Gauss–Newton method. Both methods were implemented using the same programming language (MATLAB). The database which was used in the experiments was collected by capturing a number of CT and fluoroscopy images from the bones of three knee cadavers. The data capturing processes is explained in more detail in  3.1.

### Accuracy test

In order to evaluate medical registration methods, in most studies in the literature, an accuracy measure is computed. However, ground truth position data should be known first, and then the output of the proposed registration method which is going to be evaluated can be compared with the known ground truth. The ground truth can be computed in different ways. Most researchers performed experiments using human cadavers. Images from human cadavers can provide information and data which are close to the typical patients’ images to be registered. Although some researchers used synthetic data in their evaluation experiments [20], a number of differences can be seen between real and synthetic images including object appearance, background, artefacts and noise. To evaluate the accuracy of the proposed method, we used a mechanical positioning system to collect data and to compute the ground truth from three human knee cadavers. Then, we evaluated the proposed method’s accuracy by running the method on the acquired cadaveric data and comparing the results with the gold standard. This is explained in more detail in Sec9 and Sec10.

**Table 1:** Mean error, standard deviation of error and mean absolute error of the proposed method for the relational kinematic parameters

Relational kinematic parameters	The proposed method		
	Mean error	Standard deviation of error	Mean absolute error
Flexion–extension (mm)	0.1153	0.6985	0.6195
Internal–external (mm)	$-$ .0698	0.4151	0.3426
Abduction–adduction (mm)	0.1622	0.6426	0.5750
Medial–lateral ($^{\circ }$)	0.5410	2.0432	1.7187
Anterior–posterior ($^{\circ }$)	$-$ 0.0548	1.0196	0.7254
Distraction–compression ($^{\circ }$)	0.2533	0.1986	0.2690

**Table 2:** Mean error, standard deviation of error and mean absolute error of the SCV method for the relational kinematic parameters, respectively

Relational kinematic parameters	The SCV method		
	Mean error	Standard deviation of error	Mean absolute error
Flexion–extension (mm)	0.1427	0.6611	0.5839
Internal–external (mm)	$-$ 0.0617	0.4182	0.3438
Abduction–adduction (mm)	0.1859	0.6134	0.5481
Medial–lateral ($^{\circ }$)	0.5375	2.0192	1.6864
Anterior–posterior ($^{\circ }$)	$-$ 0.0527	0.9909	0.6963
Distraction–compression ($^{\circ }$)	0.2621	0.1955	0.2752

#### Cadaveric data collection procedure

In this section, the method used for providing the cadaveric dataset with its ground truth is explained. The ground truth includes the kinematic parameters (three relational translation kinematic parameters: medial–lateral shift, anterior–posterior draw and distraction–compression, as well as the three relational rotation parameters: flexion–extension, internal–external rotation and abduction–adduction) which are most commonly used to describe knee joint movements and investigate joint kinematics [8].

Three cadaveric lower limbs supplied, with permission, by the Australian National University (ANU) medical school were used. We also used a mechanical positioning system which is shown in Fig. 2 to place the cadavers in known positions at the time of the image capturing process. The steps in the experimental procedure for each cadaver are explained below: Firstly, the cadaver was tightly attached to a wooden board. The tibia and femur were fixed tightly to the board to eliminate any relative movement between them. After that, a CT image was acquired from the fixed cadaver. The relative position of the bones, when the CT scan image was captured, was considered to be the ground truth. This means that finding the kinematic parameters when both bones (the femur and the tibia) have not moved relative to each other (i.e., when $T_x$, $T_y$, $T_z$ and $R_x$, $R_y$, $R_z$ are all zero) will produce the kinematic parameters for the bones when the CT is captured. In the next step, as can be seen in Fig. 2, the fixed cadaver was mounted using a mechanical positioning system. There are three micrometres on the system by which the position of the cadaver can be changed. In the data capturing process, these micrometres were used to change out-of-plane parameters ($R_X$, $R_Y$ and $T_Z$). Then, a number of fluoroscopy frames from each cadaveric knee were captured when the knee was in 27 different positions. Each time, when the position of the cadaver was set, the changes in rotations and the translation were a combination of the below values: $R_x$ : − 5, 0, 5 $R_y$ : − 5, 0, 5 $T_z$ : − 20, − 10, 0.

**Fig. 3:**
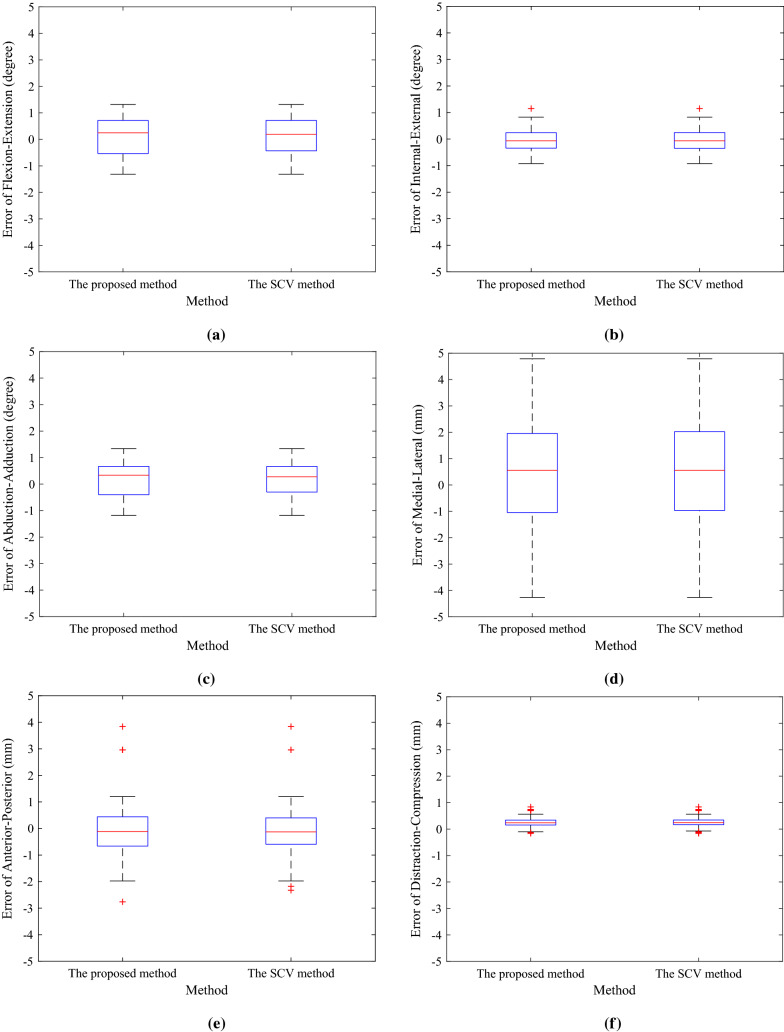
**a**, **b** and **c** show box plots of the error in relational rotation kinematic parameters per frame per cadaver obtained by the proposed and the SCV methods. **d**, **e** and **f** show box plots of the error in relational translation kinematic parameters obtained by the methods

#### Accuracy analysis

To evaluate the performance of our hybrid registration approach, we compared the proposed algorithm with the SCV registration approach in  [11]. The methods were run on the cadaveric data to register each 3D CT cadaveric knee image with its associated 27 fluoroscopy images. Then, after registration of the CT image with the fluoroscopy frames, the kinematic parameters are computed and compared with the ground truth.

The mean error, standard deviation (SD) of error and mean absolute error of the relational kinematics parameters computed by the methods are shown in Tables 1 and  2. As can be seen the mean error ± SD of the proposed method for the medial–lateral shift, anterior–posterior draw, distraction–compression, flexion–extension, internal–external rotation and abduction–adduction were 0.1153 ± 0.6985, -.0698 ± 0.4151, 0.1622 ± 0.6426, 0.5410 ± 2.0432, − 0.0548 ± 1.0196 and 0.2533 ± 0.1986, respectively. These errors are considered to be accurate and acceptable to be used for knee kinematics analysis. Furthermore, the results for the proposed method were very similar to those of the SCV method. Figure 3 shows the error of the kinematics parameters which were computed by the SCV and the proposed methods. As can be seen, for both methods, the median of the errors is close to zero. For the proposed method, the median of the errors of flexion–extension, internal–external, abduction–adduction, medial–lateral, anterior–posterior and distraction–compression was 0.2450, 0.1256, − 0.0770, 0.0183, − 0.0563 and 0.1890, respectively. For the SCV method, the results were 0.1954, 0.1433, − 0.0407, − 0.0062, − 0.0532 and 0.2043. The box plots of the errors in the relational kinematics parameters of the proposed method show that it could provide results that were almost identical to the SCV registration method. For both methods, the largest error is related to the medial–lateral kinematic parameter. The main reason for this larger error is that finding $T_z$ is much more challenging compared to the other transformation parameters as a small displacement in the direction will cause a large error. However, this error is acceptable in some applications as large translational motion between the femur and tibia in the medial–lateral direction is prevented by certain physical constraints [9]. Indeed, fibrous capsule, ligaments and muscles restrict the three relational translation kinematic parameters, medial–lateral shift, anterior–posterior draw and distraction–compression significantly. However, the greatest range of motion is for the relational rotation parameter flexion–extension while the other relational rotation parameters abduction–adduction and the internal–external rotation are also more restricted [1].

### Computational times

In certain applications, the importance of the speed of the registration method applied can be seen more clearly, for example, in the applications used for knee joint analysis, when a large number of fluoroscopy frames, for instance 300 frames, should be registered with a CT or a 3D model of the femur and tibia to provide 3D kinematic data. Although some registration methods may apply a GPU implementation based on a special workstation to reduce the execution time, they may not be applicable in standard clinics or research departments. To show the speed of the proposed method, the computational time required to register each frame for each bone using our proposed method and the SCV technique is shown in Table 3. The results show that the proposed method’s computational time is around 48.8 s, and it is almost 3.5 times faster than the SCV method while it is as accurate as the latter. In the proposed method, performing a coarse registration based on the EPD similarity measure, which is computationally efficient, helps to reduce the data to be processed in the final registration step, leading to a faster registration method.

**Table 3:** Computation Time

Method	Computation time (seconds)
The SCV method	172.5
The proposed method	48.8

## Conclusions

In medical image analysis, multimodal image registration can be very helpful in improving the accuracy of diagnosis and providing targeted treatment. While for mono-modal registration, the images to be registered are acquired by the same sensor, for multimodal image registration, the images can be taken from different devices or imaging protocols, which makes the registration process much more challenging. There are a number of challenges in multimodal image registration including, computation time and accuracy. In this paper, we proposed a hybrid multimodal registration method, which is based on the multimodal EPD and SCV similarity measures, to perform a trade-off between accuracy and computational time. The experimental results show that the proposed method is several times faster than the most recent registration methods while it maintains very similar accuracy.
